# No evidence of rapid reversibility of tenofovir alafenamide and/or integrase strand transfer inhibitor-associated weight gain

**DOI:** 10.1097/QAD.0000000000003654

**Published:** 2023-07-26

**Authors:** Myrthe L. Verburgh, Ferdinand W.N.M. Wit, Anders Boyd, Peter Reiss, Marc Van der Valk

**Affiliations:** a Department of Infectious Diseases, Amsterdam UMC location University of Amsterdam; b Division Infectious Diseases, Amsterdam Institute for Infection and Immunity; c Division Global Health, Amsterdam Public Health; d Amsterdam Institute for Global Health and Development; e HIV Monitoring Foundation; f Department of Infectious Diseases, Public Health Service of Amsterdam; g Global Health, Amsterdam UMC location University of Amsterdam, Amsterdam, The Netherlands.

**Keywords:** antiretroviral therapy switch, integrase strand transfer inhibitor, reversibility, tenofovir alafenamide, weight change

## Abstract

**Objective:**

We aimed to determine the reversibility of at least 7% weight gain within 12 months following tenofovir alafenamide (TAF) and/or integrase strand transfer inhibitor (INSTI) discontinuation in people with HIV (PWH) from the Dutch ATHENA cohort.

**Design and methods:**

PWH with at least 7% weight gain within 24 months after first switch to TAF and/or INSTI whilst being virally suppressed were selected, excluding those with comorbidities/co-medication known to be associated with weight gain. PWH who discontinued only TAF, only INSTI or TAF+INSTI, with available follow-up weight, were included. Mean weight change in the 24 months prior to and 12 months after discontinuation was modelled using mixed-effects linear regression. Factors associated with yearly weight change were assessed using linear regression.

**Results:**

In 115 PWH, discontinuing only TAF (*n* = 39), only INSTI (*n* = 53) or TAF+INSTI (*n* = 23), the adjusted mean modelled weight change in the 24 months prior to discontinuation was +4.50 kg [95% confidence interval (CI) 3.04–6.10], +4.80 kg (95% CI 2.43–7.03) and +4.13 kg (95% CI 1.50–7.13), respectively, and −1.89 kg (95% CI −3.40 to −0.37), −1.93 kg (95% CI −3.92 to +0.07) and −2.55 kg (95% CI −5.80 to +0.02) in the 12 months postdiscontinuation. A greater number of years since HIV diagnosis was associated with greater reversibility of weight gain. No associations were found between weight change postdiscontinuation and changes in NRTI backbone or anchor agent at moment of discontinuation.

**Conclusion:**

There was no evidence of rapid reversibility of at least 7% TAF-associated and/or INSTI-associated weight gain after discontinuation of these agents. Studies of larger and more diverse populations of PWH are required to more fully understand the degree to which weight gain is reversible when discontinuing TAF and/or INSTI.

## Introduction

An increasing number of studies report excessive (i.e. ≥7 or ≥10%) weight gain in both treatment-naive and virally suppressed people with HIV (PWH) after commencing antiretroviral therapy (ART) that includes tenofovir alafenamide (TAF) and/or an integrase strand transfer inhibitor (INSTI) [1–3]. We previously reported an at least 7% weight gain in 22% of virally suppressed participants from the Dutch ATHENA HIV cohort after switching to TAF-containing and/or INSTI-containing ART [4].

Small case reports suggest that TAF-associated and/or INSTI-associated weight gain seems partly reversible [5–7]. We aimed to determine the reversibility of at least 7% TAF-associated and/or INSTI-associated weight gain following discontinuation of these agents in virally suppressed PWH.

## Methods

### The AIDS Therapy Evaluation in the Netherlands cohort

Data on demographics, ART, and clinically relevant characteristics from PWH in care in the Netherlands has been prospectively collected from 1998 onward, as part of the AIDS Therapy Evaluation in the Netherlands (ATHENA) cohort [8]. People entering HIV care are counselled and receive written material about participation in the ATHENA cohort, following which they can consent verbally or elect to opt-out. Data are pseudonymized and may be used for scientific purposes. A designated data protection officer safeguards compliance with the European General Data Protection Regulation. Data collection is continuous and all data until June 2023 were used for the current analysis.

At its inception, the ATHENA cohort was approved by the institutional review boards of all participating HIV treatment centers. For this analysis, only existing data have been used and, therefore, no additional review or consent was required.

### Selection of study population

ART-experienced adults with at least 7% weight gain within 24 months after switch to TAF and/or INSTI were selected as described previously [4] (for details see Text S1). Subsequently, we identified participants who discontinued only TAF, only INSTI or both TAF+INSTI, with at least one weight measurement at least 3 months after discontinuation. We excluded participants who at the moment of discontinuing TAF and/or INSTI had less than 7% weight gain.

### Data collection

Demographic and HIV-specific characteristics included sex at birth, date of birth, region of origin, date of HIV diagnosis and date of first ART initiation. Data from routine care included height, weight, CD4^+^/CD8^+^ cell count, plasma HIV-1 RNA, any changes in ART, co-medication and incident comorbidities.

### Statistical analysis

Participants were divided in three groups: discontinuing only TAF, only INSTI or TAF+INSTI. Follow-up began at the moment of discontinuing and continued until date of pregnancy; virological failure; use of corticosteroids/antidepressants/antipsychotics; (re-)starting TAF and/or INSTI; last available weight measurement; death or 12 months, whichever occurred first.

Mean weight changes postdiscontinuation were modelled over discrete time intervals of 6 months (modelled using restricted cubic splines with five knots) using mixed-effect linear regression, with a random intercept for individuals and random slope for time. Mean weight changes were adjusted for sex, region of origin, age and last available weight prior to discontinuation. Adjusted mean modelled weight change at 24 months prior to and at 12 months after discontinuation was based on predicted values from the models. We tested whether the adjusted mean modelled weight change at 12 months postdiscontinuation was not equal to zero using a one-sample *t* test and whether this weight change was different between groups using a two-sample *t* test. In a sensitivity analysis, adjusted mean modelled weight change prior to and after discontinuation was plotted when stratified by change in TDF use at the moment of TAF and/or INSTI discontinuation.

Changes in BMI category over time were visualized using alluvial plots.

The mean weight change in kilograms per year postdiscontinuation was calculated from a linear regression model fit to each individual. Demographic, HIV-specific and ART-specific factors associated with yearly weight change were assessed using linear regression. A multivariable model was built by including all variables associated with *P* < 0.20 in univariable analyses and subsequently removing those with *P* ≥ 0.05 in backwards-stepwise fashion.

Statistical significance was defined as two-sided *P* < 0.05. Missing data were imputed using last observation carried forward. Stata/IC v15.1 (College Station, Texas, USA) and R v4.1.1 (Vienna, Austria) were used for statistical analyses.

## Results

### Study population

A total of 8304 individuals switched to a first TAF-based and/or INSTI-based regimen between May 2007 and November 2022, of whom we excluded 1718 (Figure S1). Six thousand five hundred and eighty-six participants remained of whom 2048 switched to TAF, 3153 to INSTI and 1385 to TAF+INSTI. Some 26.5% of participants switching to TAF+INSTI gained at least 7% weight within 24 months, compared with 22.1% and 22.2% in those switching to only TAF (*P* = 0.004) and only INSTI (*P* = 0.002), respectively.

Of the 1512 participants with a first recording of at least 7% weight gain, 214 participants subsequently discontinued TAF and/or INSTI (Table S1). Of those 214 of whom 115 were included, 39 discontinued only TAF; 53 only INSTI and 23 TAF+INSTI (Figure S1). Demographics of the included participants were similar to all 1512 participants with at least 7% weight gain (Table S2).

Median time between start TAF/INSTI and first recording of at least 7% weight gain was 12 months (IQR 6–18) and time between first recording of at least 7% weight gain and discontinuation of TAF and/or INSTI was 18 months (IQR 6–30). This did not differ between groups, when stratified by discontinued agent (Table 1).

**Table 1:** Characteristics of 115 participants discontinuing tenofovir alafenamide and/or integrase strand transfer inhibitor, stratified by agent(s) being discontinued.

	Discontinuing only TAF (*n* = 39)	Discontinuing only INSTI (*n* = 53)	Discontinuing both TAF+INSTI (*n* = 23)	*P*
Male sex	26 (66.7%)	41 (77.4%)	19 (82.6%)	0.38^f^
Age (years)^a^	46.5 (39.5–54.3)	50.1 (41.4–59.3)	44.1 (41.8–52.7)	0.42^g^
Region of origin				0.54^f^
Western regions	25 (64.1%)	34 (64.2%)	11 (47.8%)	
Sub-Saharan Africa	6 (15.4%)	5 (9.4%)	2 (8.7%)	
Latin America and the Caribbean	3 (7.7%)	8 (15.1%)	6 (26.1%)	
East and Southeast Asia	4 (10.3%)	3 (5.7%)	3 (13.0%)	
Other regions	1 (2.6%)	3 (5.7%)	1 (4.4%)	
Years since HIV diagnosis^a^	14.4 (8.7–17.9)	11.8 (8.0–17.0)	15.1 (10.9–18.8)	0.18^g^
Years since start of first ART^a^	11.9 (7.8–16.6)	9.8 (7.0–15.6)	11.5 (9.7–15.5)	0.35^f^
Current CD4^+^ cell count^b^	700 (490–900)	670 (460–921)	712 (511–880)	0.88^g^
Current CD8^+^ cell count^b,c^	875 (574–1174)	755 (548–888)	934 (678–1154)	0.084^g^
Current CD4^+^/CD8^+^ ratio^b,d^	0.81 (0.64–1.16)	0.92 (0.60–1.27)	0.86 (0.56–1.13)	0.49^g^
TAF regimen and/or INSTI regimen prior to discontinuation of TAF/INSTI				NA
Only TAF	33 (84.6%)	NA	NA	
Only RAL	NA	12 (22.6%)	NA	
Only EVG	NA	3 (5.7%)	NA	
Only DTG	NA	31 (58.4%)	NA	
TAF+EVG	2 (5.1%)	3 (5.7%)	12 (52.2%)	
TAF+DTG	3 (7.7%)	3 (5.7%)	0 (0.0%)	
TAF+BIC	1 (2.6%)	1 (1.9%)	11 (47.8%)	
Change in NRTI backbone when discontinuing TAF/INSTI				NA
TAF to TDF	30 (76.9%)	NA	18 (78.3%)	
TAF to ABC	0 (0.0%)	NA	0 (0.0%)	
TAF to other	9 (23.1%)	NA	5 (21.7%)	
ABC to TDF	NA	8 (15.1%)	NA	
ABC to other	NA	5 (9.4%)	NA	
Other to TDF	NA	5 (9.4%)	NA	
Other to ABC	NA	1 (1.9%)	NA	
Continue TAF	NA	7 (13.2%)	NA	
No change in NRTI backbone	NA	27 (50.9%)	NA	
Change in anchor agent when discontinuing TAF/INSTI				NA
INSTI to EFV	NA	4 (7.6%)	1 (4.4%)	
INSTI to NNRTI (other than EFV)	NA	25 (47.2%)	19 (82.6%)	
INSTI to PI	NA	21 (39.6%)	1 (4.4%)	
INSTI to other	NA	3 (5.7%)	2 (8.7%)	
PI to NNRTI (other than EFV)	1 (2.6%)	NA	NA	
PI to other	3 (7.7%)	NA	NA	
NNRTI (other than EFV) to PI	1 (2.6%)	NA	NA	
Continue INSTI	6 (15.4%)	NA	NA	
No change in anchor agent	28 (71.8%)	NA	NA	
Time between start TAF/INSTI and first recording of ≥7% WG, (months)	12 (6–18)	12 (6–12)	12 (6–12)	0.18^g^
Time between first recording of ≥7% WG and discontinuation of TAF/INSTI (months)	18 (6–30)	12 (6–30)	24 (12–36)	0.13^g^
Follow-up after discontinuation of TAF/INSTI (months)	12 (6–12)	12 (6–12)	12 (6–12)	0.34^g^
Weight at start TAF/INSTI (kg)	72.0 (63.0–85.2)	74.0 (66.0–81.7)	69.4 (61.2–81.0)	0.76^g^
BMI category at start TAF/INSTI^e^				0.92^f^
Underweight	1 (2.6%)	4 (7.6%)	2 (8.7%)	
Normal weight	26 (66.7%)	34 (64.2%)	14 (60.9%)	
Overweight	7 (18.0%)	9 (17.0%)	3 (13.0%)	
Obese	5 (12.8%)	6 (11.3%)	4 (17.4%)	
Weight at first recording of ≥7% WG (kg)	82.0 (70.0–94.0)	84.0 (72.5–90.0)	76.4 (65.5–90.0)	0.77^g^
BMI category at first recording of ≥7% WG^e^				0.39^f^
Underweight	0 (0.0%)	0 (0.0%)	0 (0.0%)	
Normal weight	12 (30.8%)	27 (50.9%)	9 (39.1%)	
Overweight	19 (48.7%)	17 (32.1%)	9 (39.1%)	
Obese	8 (20.5%)	9 (17.0%)	5 (21.7%)	
Weight at discontinuation of TAF/INSTI (kg)	82.9 (73.4–96.0)	86.0 (74.4–92.0)	79.9 (71.2–95.0)	0.72^g^
BMI category at discontinuation of TAF/INSTI^e^				0.83^f^
Underweight	0 (0.0%)	1 (1.9%)	0 (0.0%)	
Normal weight	11 (28.2%)	20 (37.7%)	8 (34.8%)	
Overweight	20 (51.3%)	19 (35.9%)	10 (43.5%)	
Obese	8 (20.5%)	13 (24.5%)	5 (21.7%)	
Absolute weight gain from start TAF/INSTI until discontinuation of TAF/INSTI (kg)	8.0 (6.4–11.0)	9.1 (7.8–13.5)	9.5 (7.5–11.0)	0.21^g^
Weight at end of follow-up (kg)	81.4 (71.2–92.0)	84.0 (70.0–91.5)	79.9 (69.8–89.2)	0.81^f^
BMI category at end of follow-up^e^				0.98^f^
Underweight	1 (2.6%)	1 (1.9%)	0 (0.0%)	
Normal weight	12 (30.8%)	20 (37.7%)	9 (39.1%)	
Overweight	19 (48.7%)	23 (43.4%)	9 (39.1%)	
Obese	7 (18.0%)	9 (17.0%)	5 (21.7%)	

ABC, abacavir; ART, antiretroviral therapy; BIC, bictegravir; DTG, dolutegravir; EFV, efavirenz; EVG, elvitegravir; INSTI, integrase strand transfer inhibitor; NNRTI, nonnucleoside reverse transcriptase inhibitor; *P*, *P* value; PI, protease inhibitor; RAL, raltegravir; TAF, tenofovir alafenamide; TDF, tenofovir disproxil fumarate; WG, weight gain. Values no. (%) or median (IQR).

a At moment of discontinuation TAF/INSTI.

b Last known value prior to moment of discontinuation.

c Current CD8^+^ cell count missing in 3/39; 5/53; and 2/23, respectively.

d Current CD4+/CD8^+^ ratio missing in 3/39; 3/53; and 1/23, respectively.

e BMI was categorized as underweight (<18.5 kg/m^2^), normal weight (18.5–24.9 kg/m^2^), overweight (25–29.9 kg/m^2^) and obese (≥30 kg/m^2^).

f Fisher's exact test.

g Kruskal–Wallis test.

Most participants were male (74.8%), of western origin (60.9%), with a median age of 46 years and known with HIV for a median of 11.8 years, and similar between the three groups. Six participants in the TAF group continued using an INSTI, and seven participants in the INSTI group continued using TAF. Details of additional ART changes at the time of discontinuing TAF/and or INSTI are summarized in Table 1.

Reasons for discontinuing TAF and/or INSTI were diverse, with weight gain specifically reported in only nine participants (7.8%) (Table S3).

### Weight change prior to and after tenofovir alafenamide discontinuation and/or integrase strand transfer inhibitor discontinuation

**Fig. 1:**
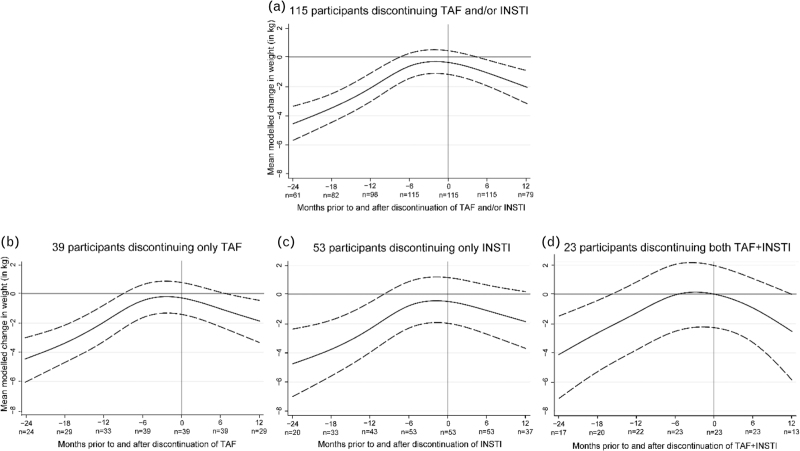
Adjusted mean modelled weight change in the 24 months prior to and 12 months after discontinuation of tenofovir alafenamide and/or integrase strand transfer inhibitor in 115 participants, stratified by agent(s) being discontinued.

The adjusted mean modelled weight change whilst on TAF and/or INSTI in the 24 months prior to discontinuation for all participants was +4.57 kg (95% CI 3.37–5.71), and −2.07 kg (95% CI −3.22 to −0.91) in the 12 months after discontinuation, representing a significant postdiscontinuation weight change (*P* < 0.001) (Fig. 1a).

The adjusted mean modelled weight change was plotted in the 24 months prior to and 12 months after discontinuation in all 115 participants (panel a); discontinuation of only TAF in 39 participants (panel b); discontinuation of only INSTI in 53 participants (panel c); and discontinuation of both TAF and INSTI in 23 participants (panel d). Time prior to discontinuation was restricted to time whilst using TAF and/or INSTI. Modelled mean weight changes in kilograms with 95% confidence interval (dotted lines) were adjusted for sex, region of origin, age at moment of discontinuation, and last available weight prior to discontinuation. INSTI, integrase strand transfer inhibitor; TFA, tenofovir alafenamide.

When stratified by the discontinued agent, participants who, respectively, discontinued only TAF, only INSTI or TAF+INSTI had an adjusted mean modelled weight change of +4.50 kg (95% CI 3.04–6.10), +4.80 kg (95% CI 2.43–7.03) and +4.13 kg (95% CI 1.50–7.13) in the 24 months prior to discontinuation, and of −1.89 kg (95% CI −3.40 to −0.37) (*P* = 0.016), −1.93 kg (95% CI −3.92 to +0.07) (*P* = 0.058) and −2.55 kg (95% CI −5.80 to +0.02) (*P* = 0.11) in the 12 months postdiscontinuation (Fig. 1b–d). Weight change postdiscontinuation was not significantly different between groups (only TAF vs. only INSTI *P* = 0.98; only TAF vs. both *P* = 0.66; only INSTI vs. both *P* = 0.74).

In a sensitivity analysis, the adjusted mean modelled weight change was plotted, stratified by concomitant change to/continued use of TDF at the moment of TAF discontinuation and/or INSTI discontinuation (Figure S2). Weight change postdiscontinuation was not significantly different between these groups (switch to TDF vs. continue TDF *P* = 0.96; switch to TDF vs. non-TDF *P* = 0.70; continue TDF vs. non-TDF *P* = 0.74).

### Changes in BMI category prior to and after tenofovir alafenamide discontinuation and/or integrase strand transfer inhibitor discontinuation

Changes in BMI category over time were similar between those discontinuing only TAF, only INSTI and TAF+INSTI (Table 1). BMI category changes over time in all 115 participants are visualized in Figure S3.

### Factors associated with weight loss after tenofovir alafenamide and/or integrase strand transfer inhibitor discontinuation

Only a greater number of years since HIV diagnosis was independently associated with more weight loss postdiscontinuation. Changes in NRTI backbone or anchor agent at the moment of TAF and/or INSTI discontinuation were not independently associated with subsequent weight change (Table S4).

## Discussion

Whether the weight gain that some PWH experience on TAF-containing and/or INSTI-containing regimens is rapidly reversible when either or both of these agents are discontinued remains unclear and is currently not recommended by guidelines [9]. In our cohort of virally suppressed PWH with at least 7% weight gain whilst on TAF-containing and/or INSTI-containing ART, individuals who discontinued only TAF, only INSTI or TAF+INSTI demonstrated similar moderate weight loss in the subsequent 12 months.

Reductions in BMI following discontinuation of TAF and/or INSTI were limited and less impressive than the BMI increases, which had been observed whilst on TAF and/or INSTI.

Starting TAF+INSTI, in both virally suppressed and ART-naive individuals, has been associated with greater weight gain than when including either one of these agents in the regimen [4,10]. In our analysis, significantly more participants switching to TAF+INSTI gained at least 7% weight compared with those switching to only TAF or only INSTI. One might, therefore, have expected weight loss to likewise be more pronounced when simultaneously discontinuing both agents. We, however, did not find a significant difference in weight change postdiscontinuation between those discontinuing both TAF+INSTI, compared with those discontinuing only TAF or only INSTI.

Taken together, our findings suggest that in PWH who experience significant increases in weight on TAF and/or INSTI, discontinuing these drugs certainly does not completely reverse these increases over the short-term. Whether this implies that some of the potential effect of TAF and/or INSTI may be irreversible needs further investigation.

Our findings are supported by data from the CHARACTERISE study, in which a subset of participants originally randomized to either TAF/FTC/DTG, TDF/FTC/DTG or TDF/FTC/EFV as part of the ADVANCE study [10] were all switched to TDF/3TC/DTG showing only modest decreases in weight after this switch [11].

We did not observe significant associations between concomitant changes in the NRTI backbone or anchor drug, including switches to TDF or EFV, and weight change following TAF discontinuation and/or INSTI discontinuation. In a sensitivity analysis, weight loss was not significantly different between those switching to TDF and continuing TDF after discontinuation of TAF and/or INSTI. However, caution is needed when interpreting the lack of such an association given our limited sample size and it likely being biased by the fact that most of the individuals (77.4%) who discontinued TAF switched to TDF at the same time, and that those continuing TDF (20/115) by definition included participants who only discontinued an INSTI.

Our study is the first to assess the reversibility of clinically significant TAF and/or INSTI-associated weight gain in a cohort of PWH discontinuing either or both these agents whilst applying strict exclusion and censoring criteria to reduce confounding by co-medication or comorbidities known to potentially affect weight. Furthermore, by only including individuals with a substantial weight gain of at least 7%, our results focus on those who might benefit most from weight-reducing interventions.

A limitation of our study is its limited sample size and lack of statistical power for some of the analyses. This includes a low number of women, which excluded any sex-specific analyses. Moreover, participants received a variety of ART regimens and were also switched to different ART regimens, resulting in a heterogeneous group. Furthermore, weight was measured in a nonstandardized manner as part of routine clinical assessments, and we were unable to account for the possible contribution of any changes in lifestyle, including changes in smoking behaviour, which might have occurred in conjunction with TAF discontinuation and/or INSTI discontinuation. Although the documented reason for discontinuing TAF and/or INSTI was weight-related in only 7.8% of participants, we cannot rule out that healthcare providers recommended lifestyle changes in any individual with an at least 7% weight gain.

In conclusion, at least 7% weight gain associated with TAF and/or INSTI appears to be incompletely reversible in the short-term with only minor improvements in BMI. Additional research with larger and diverse populations of PWH is required to validate and better understand the reversibility of this weight gain upon discontinuation of TAF and/or INSTI, including the contribution of including potentially weight-suppressing antiretroviral drugs in the subsequent regimen. Additional studies are required to better understand the causes of TAF-associated and/or INSTI-associated weight gain and develop targeted strategies to prevent it.

## Acknowledgements

**Clinical centers (**^**∗**^**denotes site coordinating physician)**.

Amsterdam UMC, AMC site, Amsterdam: HIV treating physicians: M. van der Valk^∗^, S.E. Geerlings, A. Goorhuis, V.C. Harris, J.W. Hovius, B. Lempkes, F.J.B. Nellen, T. van der Poll, J.M. Prins, P. Reiss, V. Spoorenberg, M. van Vugt, W.J. Wiersinga, F.W.M.N. Wit. HIV nurse consultants: C. Bruins, M. van Duinen, J. van Eden, A.M.H. van Hes, F.J.J. Pijnappel, S.Y. Smalhout, A.M. Weijsenfeld. HIV clinical virologists/ chemists: N.K.T. Back, B. Berkhout, M.T.E. Cornelissen, R. van Houdt, M. Jonges, S. Jurriaans, C.J. Schinkel, K.C. Wolthers, H.L. Zaaijer.

Amsterdam UMC, VUmc site, Amsterdam: HIV treating physicians: E.J.G. Peters^∗^, M.A. van Agtmael, R.S. Autar, M. Bomers, K.C.E. Sigaloff. HIV nurse consultants: M. Heitmuller, L.M. Laan, J. Steiner. HIV clinical virologists/chemists: N.K.T. Back, B. Berkhout, M.T.E. Cornelissen, R. van Houdt, M. Jonges, S. Jurriaans, C.J. Schinkel, K.C. Wolthers, H.L. Zaaijer.

Emma Kinderziekenhuis (Amsterdam UMC, AMC site), Amsterdam: HIV treating physicians: M. van der Kuip, D. Pajkrt. HIV nurse consultants: A.M. Weijsenfeld.

Admiraal De Ruyter Ziekenhuis, Goes: HIV treating physicians: M. van den Berge^∗^, A. Stegeman. HIV nurse consultants: S. Baas, L. Hage de Looff. HIV clinical virologists/chemists: P. van Keulen, J. Stohr, B. Wintermans.

Catharina Ziekenhuis, Eindhoven: HIV treating physicians: M.J.H. Pronk^∗^, H.S.M. Ammerlaan. HIV nurse consultants: E.S. de Munnik. HIV clinical virologists/chemists: B. Deiman, A.R. Jansz, V. Scharnhorst, J. Tjhie, M.C.A. Wegdam.

DC Klinieken Lairesse – Hiv Focus Centrum, Amsterdam: HIV treating physicians: M. van der Valk^∗^, A. van Eeden, E. Hoornenborg, J. Nellen. HIV nurse consultants: L.J.M. Elsenburg, H. Nobel. HIV clinical virologists/chemists: C.J. Schinkel.

ETZ (Elisabeth-TweeSteden Ziekenhuis), Tilburg: HIV treating physicians: M.E.E. van Kasteren^∗^, M.A.H. Berrevoets, A.E. Brouwer. HIV nurse specialist: B.A.F.M. de Kruijf-van de Wiel. HIV nurse consultants: A. Adams, A. Dievelaar-Oomen, R. van Erve, S. Phaf, B. van de Ven. HIV data collection: B.A.F.M. de Kruijf-van de Wiel. HIV clinical virologists/chemists: A.G.M. Buiting, J.L. Murck.

Erasmus MC, Rotterdam: HIV treating physicians: C. Rokx^∗^, A.A. Anas, H.I. Bax, E.C.M. van Gorp, M. de Mendonça Melo, E. van Nood, J.L. Nouwen, B.J.A. Rijnders, C.A.M. Schurink, L. Slobbe, T.E.M.S. de Vries-Sluijs. HIV nurse consultants: N. Bassant, J.E.A. van Beek, M. Vriesde, L.M. van Zonneveld. HIV data collection: J. de Groot. HIV clinical virologists/chemists: J.J.A. van Kampen, M.P.G Koopmans.

Erasmus MC Sophia Kinderziekenhuis, Rotterdam: HIV treating physicians: P.L.A. Fraaij, A.M.C. van Rossum, C.L. Vermont. HIV nurse consultants: L.C. van der Knaap.

Flevoziekenhuis, Almere: HIV treating physicians: J. Branger^∗^, R.A. Douma. HIV nurse consultant: A.S. Cents-Bosma, C.J.H.M. Duijf-van de Ven.

HagaZiekenhuis, Den Haag: HIV treating physicians: E.F. Schippers^∗^, C. van Nieuwkoop, L. Smit. HIV nurse consultants: J. Geilings, S. van Winden. HIV data collection: G. van der Hut. HIV clinical virologists/chemists: N.D. van Burgel.

HMC (Haaglanden Medisch Centrum), Den Haag: HIV treating physicians: E.M.S. Leyten^∗^, L.B.S. Gelinck, F. Mollema. HIV nurse consultants: S. Davids-Veldhuis, C. Tearno, G.S. Wildenbeest. HIV clinical virologists/chemists: E. Heikens.

Isala, Zwolle: HIV treating physicians: P.H.P. Groeneveld^∗^, J.W. Bouwhuis, A.J.J. Lammers. HIV nurse consultants: A.G.W. van Hulzen, S. Kraan, M.S.M. Kruiper. HIV data collection: G.L. van der Bliek, P.C.J. Bor. HIV clinical virologists/chemists: S.B. Debast, G.H.J. Wagenvoort.

Leids Universitair Medisch Centrum, Leiden: HIV treating physicians: A.H.E. Roukens^∗^, M.G.J. de Boer, H. Jolink, M.M.C. Lambregts, A.H.E. Roukens, H. Scheper. HIV nurse consultants: W. Dorama, N. van Holten. HIV clinical virologists/chemists: E.C.J. Claas, E. Wessels.

Maasstad Ziekenhuis, Rotterdam: HIV treating physicians: J.G. den Hollander^∗^, R. El Moussaoui, K. Pogany. HIV nurse consultants: C.J. Brouwer, D. Heida-Peters, E. Mulder, J.V. Smit, D. Struik-Kalkman.

HIV data collection: T. van Niekerk. HIV clinical virologists/chemists: O. Pontesilli, C. van Tienen.

Maastricht UMC±, Maastricht: HIV treating physicians: S.H. Lowe^∗^, A.M.L. Oude Lashof, D. Posthouwer, M.E. van Wolfswinkel. HIV nurse consultants: R.P. Ackens, K. Burgers, J. Schippers. HIV data collection: B. Weijenberg-Maes. HIV clinical virologists/chemists: T.R.A. Havenith, M. van Loo.

Medisch Centrum Leeuwarden, Leeuwarden: HIV treating physicians: M.G.A. van Vonderen^∗^, L.M. Kampschreur. HIV nurse consultants: M.C. van Broekhuizen, S, Faber. HIV clinical virologists/chemists: A. Al Moujahid.

Medisch Spectrum Twente, Enschede: HIV treating physicians: G.J. Kootstra^∗^, C.E. Delsing. HIV nurse consultants: M. van der Burg-van de Plas, L. Scheiberlich.

Noordwest Ziekenhuisgroep, Alkmaar: HIV treating physicians: W. Kortmann^∗^, G. van Twillert^∗^, R. Renckens, J. Wagenaar. HIV nurse consultants & HIV data collection: D. Ruiter-Pronk, F.A. van Truijen-Oud.

HIV clinical virologists/chemists: J.W.T. Cohen Stuart, M. Hoogewerf, W. Rozemeijer, J.C. Sinnige.

OLVG, Amsterdam: HIV treating physicians: K. Brinkman^∗^, G.E.L. van den Berk, K.D. Lettinga, M. de Regt, W.E.M. Schouten, J.E. Stalenhoef, J. Veenstra, S.M.E. Vrouenraets. HIV nurse consultants: H. Blaauw, G.F. Geerders, M.J. Kleene, M. Knapen, M. Kok, I.B. van der Meché, A.J.M. Toonen, S. Wijnands, E. Wttewaal. HIV clinical virologists: D. Kwa, T.J.W. van de Laar.

Radboudumc, Nijmegen: HIV treating physicians: R. van Crevel^∗^, K. van Aerde, A.S.M. Dofferhoff, S.S.V. Henriet, H.J.M. ter Hofstede, J. Hoogerwerf, O. Richel. HIV nurse consultants: M. Albers, K.J.T. Grintjes-Huisman, M. de Haan, M. Marneef. HIV clinical virologists/chemists: F.F. Stelma. HIV clinical pharmacology consultant: D. Burger.

Rijnstate, Arnhem: HIV treating physicians: E.H. Gisolf^∗^, M. Claassen, R.J. Hassing, HIV nurse consultants: G. ter Beest, P.H.M. van Bentum, M. Gelling, N. Langebeek. HIV clinical virologists/chemists: C.M.A. Swanink, M. Klein Velderman.

Spaarne Gasthuis, Haarlem: HIV treating physicians: S.F.L. van Lelyveld^∗^, R. Soetekouw. HIV nurse consultants: L.M.M. van der Prijt, J. van der Swaluw. HIV clinical virologists/chemists: J.S. Kalpoe, A. Vahidnia, A. Wagemakers.

Medisch Centrum Jan van Goyen, Amsterdam: HIV treating physicians: F.N. Lauw, D.W.M. Verhagen. HIV nurse consultants: M. van Wijk.

Universitair Medisch Centrum Groningen, Groningen: HIV treating physicians: W.F.W. Bierman^∗^, M. Bakker, R.A. van Bentum, M.A. van den Boomgaard, J. Kleinnijenhuis, E. Kloeze, A. Middel, D.F. Postma, Y. Stienstra, M. Wouthuyzen-Bakker. HIV nurse consultants: A. Boonstra, H. de Groot-de Jonge, M.M.M. Maerman, P.A. van der Meulen, D.A. de Weerd. HIV clinical virologists/chemists: K.J. van Eije, M. Knoester, C.C. van Leer-Buter, H.G.M. Niesters.

Beatrix Kinderziekenhuis (Universitair Medisch Centrum Groningen), Groningen: HIV treating physicians: E.H. Schölvinck, A.R. Verhage. HIV nurse consultants: H. de Groot-de Jonge. HIV clinical virologists/chemists: M. Knoester, C.C. van Leer-Buter, H.G.M. Niesters.

Universitair Medisch Centrum Utrecht, Utrecht: HIV treating physicians: T.Mudrikova^∗^, R.E. Barth, A.H.W. Bruns, P.M. Ellerbroek, M.P.M. Hensgens, J.J. Oosterheert, E.M. Schadd, A. Verbon, B.J. van Welzen. HIV nurse consultants: K. Aarsman, B.M.G. Griffioen-van Santen, I. de Kroon. HIV data collection: M. van Berkel, C.S.A.M. van Rooijen. HIV clinical virologists/chemists: L.M. Hofstra, R. Schuurman, A.M.J. Wensing.

Wilhelmina Kinderziekenhuis, UMC Utrecht, Utrecht: HIV treating physicians: S.P.M. Geelen, Y.G.T. Loeffen, T.F.W. Wolfs. HIV nurse consultants: N. Nauta.

Curaçao Medical Center, Willemstad (Curaçao): HIV treating physicians: E.O.W. Rooijakkers, D. van de Wetering. HIV nurse consultants: A. Alberto. Data collection: I. der Meer.

**Coordinating center**

Board of directors: M. van der Valk, S. Zaheri. HIV data analysis: A.C. Boyd, D.O. Bezemer, A.I. van Sighem, C. Smit, F.W.M.N. Wit. Data HIV data management and quality control: M.M.J. Hillebregt, T.J. Woudstra, T. Rutkens. HIV data monitoring: D. Bergsma, N.M. Brétin, K.J. Lelivelt, L. van de Sande, K.M. Visser, S.T. van der Vliet. HIV data collection: F. Paling, L.G.M. de Groot-Berndsen, M. van den Akker, R. Alexander, Y. Bakker, A. El Berkaoui, M. Bezemer-Goedhart, E.A. Djoechro, M. Groters, L.E. Koster, C.R.E. Lodewijk, R.J. Loenen, E.G.A. Lucas, S. van Meerveld, L. Munjishvili, B.M. Peeck, C.M.J. Ree, R. Regtop, A.F. van Rijk, Y.M.C. Ruijs-Tiggelman, P.P. Schnörr, M.J.C. Schoorl, E.M Tuijn, D.P. Veenenberg, E.C.M Witte. Patient registration: Y.M.C. Ruijs-Tiggelman, D. Bergsma.

Author contributions: all authors contributed to the conceptualization and/or design of the study. M.L.V., F.W.N.M.W. and A.B. contributed to methodology and formal analysis. M.L.V. verified the underlying data, performed data curation, validation, visualization and wrote the original draft of the manuscript. M.v.d.V. was responsible for funding acquisition. P.R. and M.v.d.V. supervised the study. All authors reviewed and approved the final manuscript.

Source of funding: the ATHENA cohort is managed by the HIV Monitoring Foundation (Stichting HIV Monitoring) and supported by a grant from the Dutch Ministry of Health, Welfare and Sport through the Center for Infectious Disease Control of the National Institute for Public Health and the Environment.

### Conflicts of interest

F.W.N.M.W. has served on scientific advisory boards for ViiV Healthcare and Gilead sciences. P.R. through his institution has received independent scientific grant support from Gilead Sciences, Janssen Pharmaceuticals Inc, Merck & Co and ViiV Healthcare, and has served on scientific advisory boards for Gilead Sciences, ViiV Healthcare, and Merck & Co honoraria for which were all paid to his institution. M.v.d.V. through his institution has received independent scientific grant support and consultancy fees from AbbVie, Gilead Sciences, MSD, and ViiV Healthcare, for which honoraria were all paid to his institution. M.L.V. and A.B. declare no competing interests.

## Supplementary Material

Supplemental Digital Content
