# Supplementary material for: No evidence of rapid reversibility of tenofovir alafenamide and/or integrase strand transfer inhibitor-associated weight gain
Source: AIDS. 2023 Jul 26;37(12):1843–50. doi: 10.1097/QAD.0000000000003654 (PMC10481918; doi:10.1097/QAD.0000000000003654)
Supplement: Supplemental Digital Content [file aids-37-1843-s001.docx]

**SUPPLEMENTARY DATA TO MANUSCRIPT**: “No evidence of rapid reversibility of tenofovir alafenamide and/or integrase strand transfer inhibitor-associated weight gain”

**AUTHORS**

Myrthe L. Verburgh, Ferdinand W.N.M. Wit, Anders Boyd, Peter Reiss and Marc van der Valk; on behalf of the ATHENA national observational cohort

**CONTENT**

**Text S1** …………………………………………………………………………….. page 2

**Figure S1** …………………………………………………………………………….. page 3

**Table S1** …………………………………………………………………………….. page 4

**Table S2** …………………………………………………………………………….. page 5

**Table S3** …………………………………………………………………………….. page 7

**Figure S2** …………………………………………………………………………….. page 8

**Figure S3** …………………………………………………………………………….. page 9

**Table S4**  …………………………………………………………………………….. page 11

**Supplementary Text S1 Details of methods**

Selection of study population

First, we identified ART-experienced adults (≥18 years) who switched to TAF- and/or INSTI-containing ART while virally suppressed for ≥12 months (allowing isolated HIV-1 RNA measurements <200copies/mL). Individuals with >90 days exposure to TAF and/or INSTI, ≥1 weight measurement ≤24 months prior to switching and ≥1 weight measurement ≥3 months after switching were eligible. Individuals who at the time of switch used medication (i.e., corticosteroids, antidepressants, or antipsychotics) or developed conditions known to be associated with WG (i.e., hypothyroidism, Cushing's syndrome, congestive heart failure, renal failure or liver cirrhosis) were excluded. We also excluded individuals in whom any of these conditions were diagnosed and/or these medication were started after ART-switch since these conditions could predispose individuals to WG that is unlikely to be attributed to switching ART. Females who were pregnant when switching ART were also excluded.

We subsequently selected those with ≥7% WG (relative to their weight prior to switch) within 24 months after switch to TAF and/or INSTI. In these participants, we defined follow-up beginning at the moment of first switch to TAF and/or INSTI and continuing until first recording of ≥7% WG; date of pregnancy; virological failure; use of corticosteroids, antidepressants or antipsychotics; discontinuing ART >3 months (including discontinuation of either TAF or INSTI for individuals who switched to TAF and INSTI); switching to INSTI-based ART (for those in the TAF only group); switching to TAF-containing ART (for those in the INSTI only group); last available weight measurement; death or 24 months, whichever occurred first.

**Supplementary Figure S1 Selection of ATHENA cohort participants for analysis**

Abbreviations: ART, antiretroviral therapy; INSTI, integrase strand transfer inhibitor; TAF, tenofovir alafenamide.


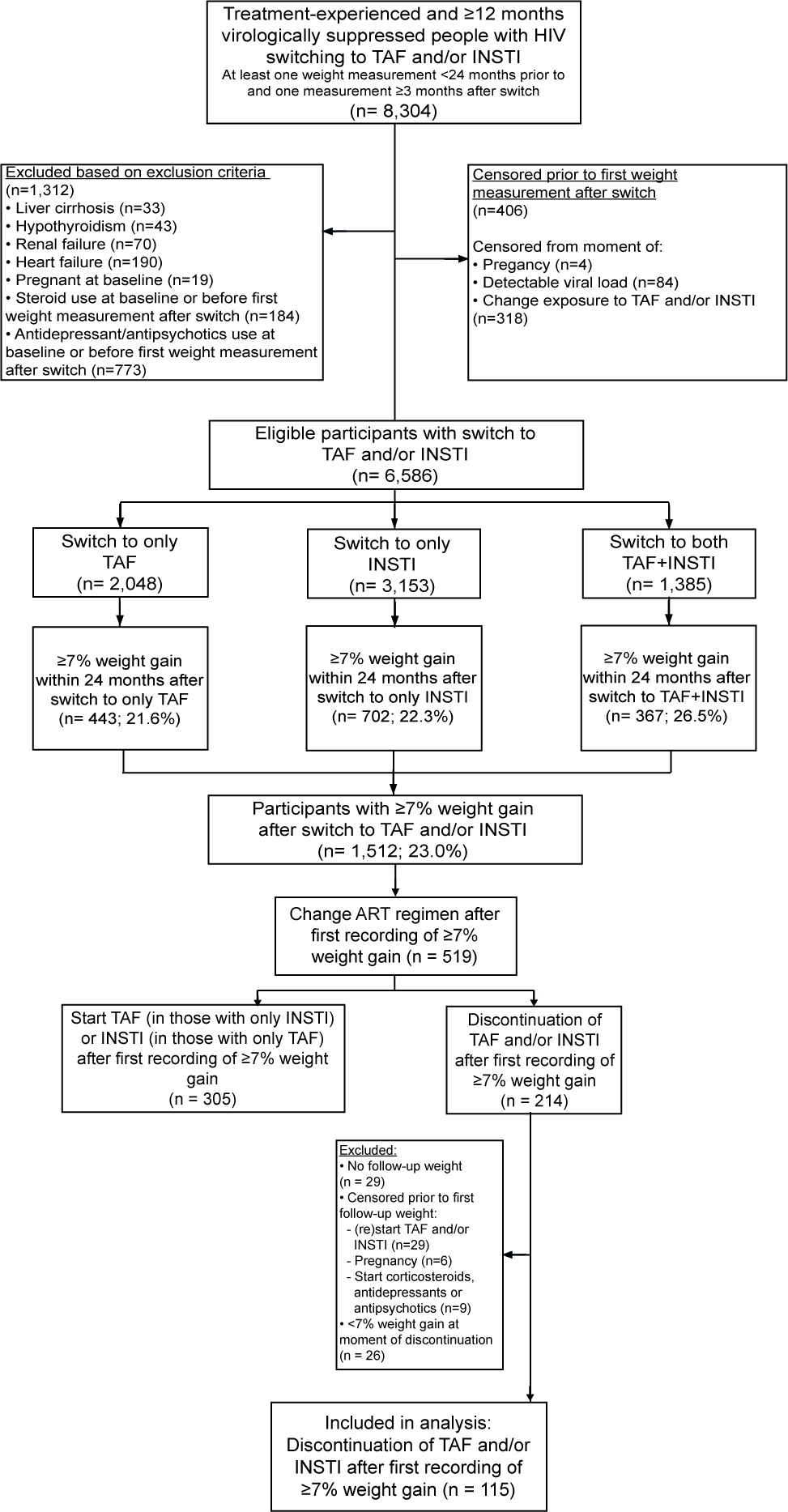


**Supplementary Table S1 Reason for discontinuation of TAF and/or INSTI in included and excluded participants discontinuing TAF and/or INSTI after first recording of ≥7% weight gain**

|  | **Total group**  **(n = 214)** | **Excluded from final analysis**  **(n = 99)** | **Included in final analysis**  **(n = 115)** |
| --- | --- | --- | --- |
| **Reason discontinuation** |  |  |  |
| Patient decision | 15 (7.0%) | 8 (8.1%) | 7 (6.1%) |
| To exclude side effects | 18 (8.4%) | 9 (9.1%) | 9 (7.8%) |
| Weight gain | 16 (7.5%) | 7 (7.1%) | 9 (7.8%) |
| Virological failure | 7 (3.3%) | 1 (1.0%) | 6 (5.2%) |
| Immunological failure | 2 (0.9%) | 0 (0.0%) | 2 (1.7%) |
| Interaction with co-medication | 6 (2.8%) | 5 (5.1%) | 1 (0.9%) |
| Cost reduction | 12 (5.6%) | 2 (2.0%) | 10 (8.7%) |
| New available ARV drugs or ARV brand to generic | 4 (1.9%) | 1 (1.0%) | 3 (2.6%) |
| By protocol (study) | 13 (6.1%) | 6 (6.1%) | 7 (6.1%) |
| Simplification | 46 (21.5%) | 24 (24.2%) | 22 (19.1%) |
| Pregnancy or pregnancy wish | 12 (5.6%) | 8 (8.1%) | 4 (3.5%) |
| Toxicity  - Sleeping problems  - Elevated lipids  - Itch / rash  - Gastro-intestinal side-effects  - Headache / dizziness  - Mood disorders (including anxiety / depression)  - Myalgia  - Other  - Not specified | 43 (20.1%)  - 6  - 6  - 1  - 7  - 5  - 10  - 1  - 6  - 1 | 15 (15.2%)  - 3  - 2  - 0  - 3  - 2  - 3  - 0  - 1  - 1 | 28 (24.3%)  - 3  - 4  - 1  - 4  - 3  - 7  - 1  - 5  - 0 |
| Other | 20 (9.3%) | 13 (13.1%) | 7 (6.1%) |

Values no. (%). Abbreviations: ARV, antiretroviral; INSTI, integrase strand transfer inhibitor; TAF, tenofovir alafenamide.

**Supplementary Table S2 Characteristics of all 1,512 participants with ≥7% weight gain after switch to TAF and/or INSTI compared to the included 115 participants discontinuing TAF and/or INSTI**

|  | **All participants with ≥7% weight gain after switch to TAF and/or INSTI (n = 1,512)** | **Included participants who discontinued TAF and/or INSTI**  **(n = 115)** |
| --- | --- | --- |
| **Male sex** | 1,181 (78.1%) | 86 (74.8%) |
| **Age,** years ^A^ | 47.9 (40.1 – 55.9) | 46.0 (39.5 – 55.1) |
| **Region of origin**  - Western regions  - Sub-Saharan Africa  - Latin America and the Caribbean  - East and Southeast Asia  - Other regions | 976 (64.7%)  210 (13.9%)  193 (12.8%)  79 (5.2%)  50 (3.3%) | 70 (60.9%)  13 (11.3%)  17 (14.8%)  10 (8.7%)  5 (4.4%) |
| **Years since HIV diagnosis** ^A^ | 11.6 (7.7 – 16.7) | 11.8 (6.9 – 16.6) |
| **Years since start of first ART** ^A^ | 9.7 (6.3 – 14.8) | 9.4 (6.2 – 14.5) |
| **Current CD4 cell count** ^B, C^ | 696 (530 – 908) | 690 (500 – 910) |
| **Current CD8 cell count** ^B, D^ | 850 (630 – 1140) | 830 (630 – 1110) |
| **CD4/8 ratio** ^B, E^ | 0.82 (0.60 – 1.14) | 0.81 (0.59 – 1.14) |
| **Time between start TAF/INSTI and first recording of ≥7% WG**, months | 12 (6 – 18) | 12 (6 – 18) |
| **Weight at start TAF/INSTI**, kg | 74.0 (64.6 – 83.0) | 72.5 (63.8 – 82.9) |
| **BMI category at start TAF/INSTI** ^F^  - Underweight  - Normal weight  - Overweight  - Obese | 75 (5.0%)  918 (60.7%)  380 (25.1%)  139 (9.2%) | 7 (6.1%)  74 (64.4%)  19 (16.5%)  15 (13.0%) |
| **Weight at first recording of ≥7% WG**, kg | 81.7 (71.6 – 91.4) | 81.0 (72.4 – 92.0) |
| **BMI category at first recording of ≥7% WG** ^F^  - Underweight  - Normal weight  - Overweight  - Obese | 17 (1.1%)  598 (39.6%)  605 (40.0%)  292 (19.3%) | 0 (0.0%)  48 (41.8%)  45 (39.1%)  22 (19.1%) |

Values no. (%) or median (IQR). Abbreviations: ART, antiretroviral therapy; BIC, bictegravir; BMI, body mass index; DTG, dolutegravir; EVG, elvitegravir; INSTI, integrase strand transfer inhibitor; NA, not applicable; P, p-value; RAL, raltegravir; TAF, tenofovir alafenamide; WG, weight gain.

A. At moment of first recording of ≥7% WG. B. Last known value prior to first recording of ≥7% WG. C. Current CD4 cell count missing in 24/1,512. D. Current CD8 cell count missing in 213/1,512; and 12/115. E. Current CD4/8 ratio missing in 182/1,512; and 8/115. F. BMI was categorized as underweight (<18.5 kg/m^2^), normal weight (18.5 to 24.9 kg/m^2^), overweight (25.0 to 29.9 kg/m^2^) and obese (≥30.0 kg/m^2^).

**Supplementary Table S3 Reason for discontinuation of TAF and/or INSTI in 115 participants, stratified by agent(s) being discontinued**

|  | **Discontinuing only TAF**  **(n = 39)** | **Discontinuing only INSTI**  **(n = 53)** | **Discontinuing both TAF+INSTI**  **(n = 23)** |
| --- | --- | --- | --- |
| **Reason discontinuation** |  |  |  |
| Patient decision | 4 (10.3%) | 2 (3.8%) | 1 (4.4%) |
| To exclude side effects | 2 (5.1%) | 4 (7.6%) | 3 (13.0%) |
| Weight gain | 3 (7.7%) | 4 (7.6%) | 2 (8.7%) |
| Virological failure | 0 (0.0%) | 5 (9.4%) | 1 (4.4%) |
| Immunological failure | 0 (0.0%) | 2 (3.8%) | 0 (0.0%) |
| Interaction with co-medication | 0 (0.0%) | 1 (1.9%) | 0 (0.0%) |
| Cost reduction | 4 (10.3%) | 0 (0.0%) | 6 (26.1%) |
| New available ARV drugs or ARV brand to generic | 2 (5.1%) | 1 (1.9%) | 0 (0.0%) |
| By protocol (study) | 0 (0.0%) | 5 (9.4%) | 2 (8.7%) |
| Simplification | 12 (30.8%) | 7 (13.2%) | 3 (13.0%) |
| Pregnancy wish | 2 (5.1%) | 2 (3.8%) | 0 (0.0%) |
| Toxicity  - Sleeping problems  - Elevated lipids  - Itch / rash  - Gastro-intestinal side-effects  - Headache / dizziness  - Mood disorders (including anxiety / depression)  - Myalgia  - Other  - Not specified | 9 (23.1%)  - 1  - 4  - 0  - 0  - 0  - 0  - 1  - 3  - 0 | 17 (32.1%)  - 2  - 0  - 1  - 4  - 3  - 6  - 0  - 1  - 0 | 2 (8.7%)  - 0  - 0  - 0  - 0  - 0  - 1  - 0  - 1  - 0 |
| Other | 1 (2.6%) | 3 (5.7%) | 3 (13.0%) |

Values no. (%). Abbreviations: ARV, antiretroviral; INSTI, integrase strand transfer inhibitor; TAF, tenofovir alafenamide.

**Supplementary Figure S2 Adjusted mean modelled weight change in the 24 months prior to and 12 months after discontinuation of TAF and/or INSTI in 115 participants, stratified by concomitant change to/continued use of TDF**

**
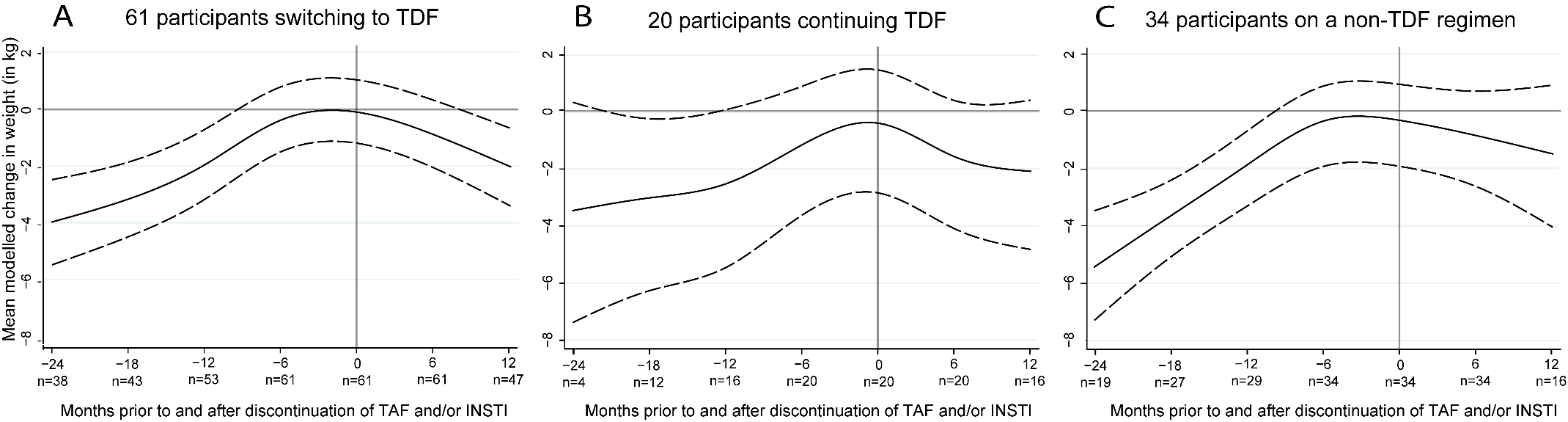
**

The adjusted mean modelled weight change was plotted in the 24 months prior to and 12 months after discontinuation of TAF and/or INSTI in 61 participants with a concomitant switch to TDF at the moment of discontinuation (panel A); in 20 participants who continued TDF at the moment of discontinuation (panel B); and in 34 participants on a non-TDF regimen prior to and after discontinuation (panel C). Time prior to discontinuation was restricted to time whilst using TAF and/or INSTI. Modelled mean weight changes in kilograms with 95% confidence interval (dotted lines) were adjusted for sex, region of origin, age at moment of discontinuation and last available weight prior to discontinuation.

Of the 115 participants, 61 switched to TDF at the moment of discontinuation, 20 continued using TDF and 34 used a non-TDF regimen prior to and after discontinuation. The adjusted mean modelled weight change was +3.97kg [95%CI, 2.49-5.48], +3.48kg [95%CI, -0.30 to +7.39] and +5.44kg [95%CI, 3.48-7.30] in these groups, respectively in the 24 months prior to discontinuation, and -2.04kg [95%CI, -3.44 to -0.63] (p=0.0054), -2.11kg [95%CI, -4.95 to +0.38] (p=0.13) and -1.50kg [-4.05 to +0.90] (p=0.25), respectively in the 12 months post-discontinuation.

**Supplementary Figure S3 Changes in BMI category in 115 participants discontinuing TAF and/or INSTI**


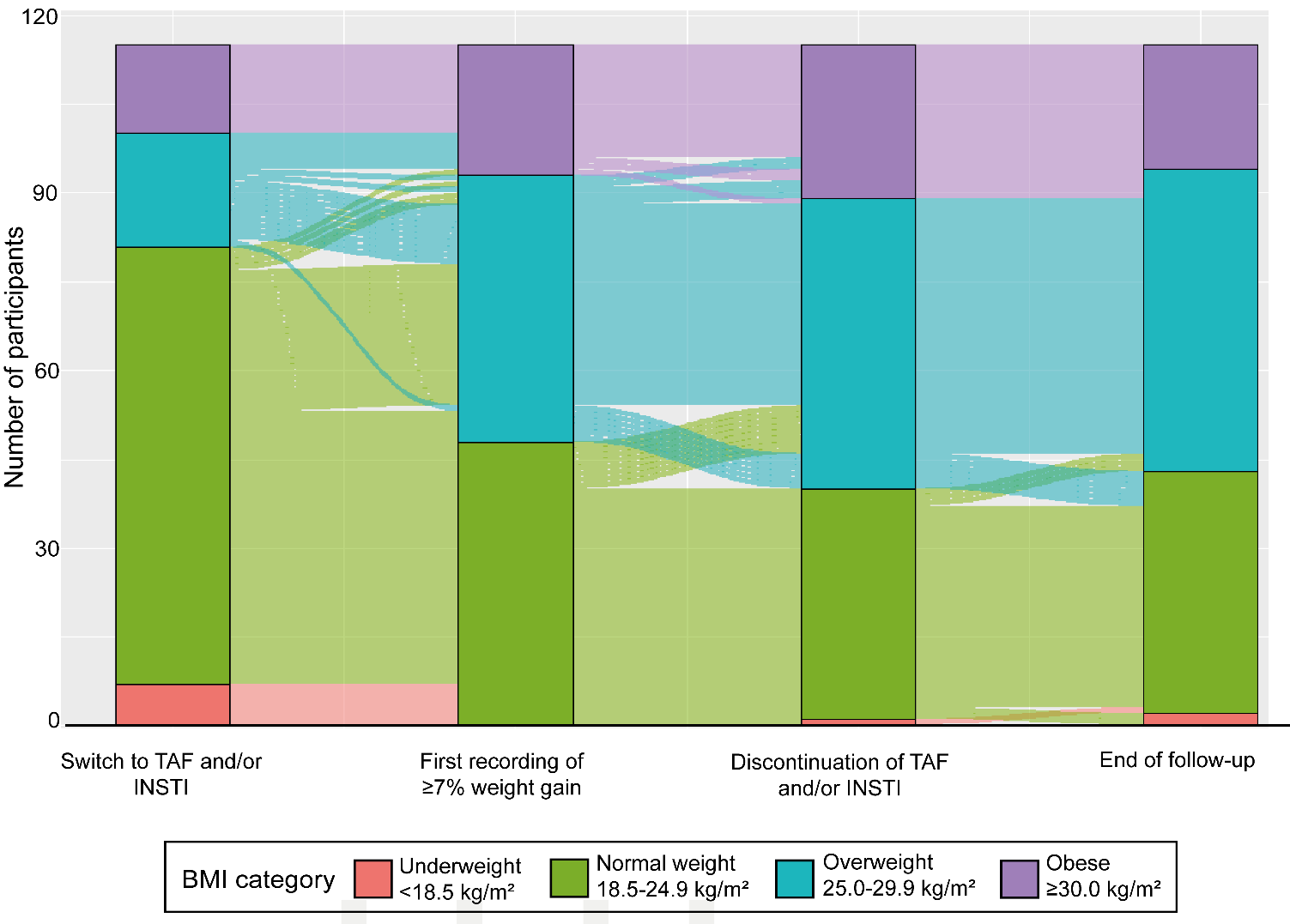


This diagram represents the observed change in BMI category in all 115 participants from switch to TAF and/or INSTI; first recording of ≥7% weight gain; discontinuation of TAF and/or INSTI; to end of follow-up after discontinuation. Bars at these time points represent the distribution of the four BMI categories. The stream fields between blocks represent changes from one BMI category to another or remaining in the same BMI category, between two time points.

Between the start of TAF and/or INSTI and the moment of discontinuation, 52.2% (60/115) had remained in the same BMI category, whereas 5.2% (n=6) had a BMI change from underweight to normal weight; 33.0% (n=38) from normal weight to overweight and 9.6% (n=11) from normal weight (n=3) or overweight (n=8) to obesity. After TAF- and/or INSTI-discontinuation, 85.2% had remained in the same BMI category at the end of follow-up. Only four participants had a further increase in weight with a change from underweight to normal weight in 0.9% (n=1) and from normal weight to overweight in 2.6% (n=3). Weight loss led to a BMI change from obesity to overweight in 4.3% (n=5); from overweight to normal weight in 5.2% (n=6); and from normal weight to underweight in 1.7% (n=2).

**Supplementary Table S4 Factors associated with mean yearly change in weight after discontinuation of TAF and/or INSTI in 115 participants**

|  | **Univariable analysis** | | **Multivariable analysis ^A^** | |
| --- | --- | --- | --- | --- |
|  | **Coeff. (95% CI)** | ***P*** | **Coeff. (95% CI)** | ***P*** |
| **Age** ^B^ *(absolute, per 10 years increase)* | -0.49 (-1.48 to +0.50) | .33 |  |  |
| **Sex**  - Male  - Female | REF  -0.59 (-3.09 to +1.91) | .64 |  |  |
| **Region of origin**  - Western regions  - Sub-Saharan Africa  - Latin America and the Caribbean  - Other (including Asian) | REF  -0.03 (-3.57 to +3.51)  -0.46 (-3.63 to +2.71)  -1.25 (-4.59 to +2.08) | .90 |  |  |
| **Weight** ^B^ *(absolute, per 1 kg increase)* | -0.03 (-0.10 to +0.04) | .41 |  |  |
| **BMI category** ^B, C^  - Normal weight  - Overweight  - Obese | REF  -1.84 (-4.28 to +0.60)  -3.06 (-5.94 to -0.17) | .098 |  |  |
| **Time between first recording of ≥7% weight gain and discontinuation of TAF/INSTI** *(absolute, per month increase)* | +0.03 (-0.03 to +0.08) | .34 |  |  |
| **Weight change prior to discontinuation** *(absolute, per 1 kg/y increase)* | +0.14 (-0.21 to +0.50) | .43 |  |  |
| **Type of INSTI prior to discontinuation**  - RAL  - EVG  - DTG  - BIC | REF  +1.13 (-3.27 to +5.53)  +1.66 (-2.35 to +5.66)  +2.47 (-2.36 to +7.29) | .76 |  |  |
| **Discontinued agent**  - Discontinuing only TAF  - Discontinuing only INSTI  - Discontinuing TAF+INSTI | REF  +0.68 (-1.79 to +3.14)  +0.98 (-2.09 to +4.05) | .79 |  |  |
| **Change in NRTI backbone when discontinuing TAF/INSTI**  - No change in NRTI backbone  - Continue TAF  - TAF to TDF  - TAF to other  - ABC to TDF  - ABC to other  - Other to TDF  - Other to ABC | REF  -2.46 (-7.17 to +2.25)  -0.40 (-3.07 to +2.28)  -1.56 (-5.22 to +2.09)  +1.57 (-2.90 to +6.04)  +5.44 (+0.03 to +10.85)  -8.05 (-13.46 to -2.64)  -0.72 (-12.03 to +10.59) | .021 |  |  |
| **Change in TDF when discontinuing TAF/INSTI**  - Switch to TDF  - Continue TDF  - Continue a non-TDF regimen | REF  +0.05 (-2.96 to +3.05)  +0.82 (-1.68 to +3.32) | .80 |  |  |
| **Change in anchor agent when discontinuing TAF/INSTI**  - No change in anchor agent  - Continue INSTI  - INSTI to EFV  - INSTI to NNRTI (other than EFV)  - INSTI to PI  - INSTI to other  - PI to NNRTI  - PI to other  - NNRTI (other than EFV) to PI | REF  -0.63 (-5.87 to +4.61)  -0.48 (-6.14 to +5.18)  +0.52 (-2.28 to +3.31)  -1.52 (-4.83 to +1.78)  -0.11 (-6.34 to +6.13)  +2.07 (-9.82 to +13.96)  -7.33 (-14.42 to -0.24)  -0.38 (-12.27 to +11.51) | .61 |  |  |
| **Years since HIV diagnosis** ^B^  *(absolute, per 10 years increase)* | -1.77 (-3.46 to -0.07) | .041 | -1.77 (-3.46 to -0.07) | .041 |
| **Years since start of first ART** ^B^  *(absolute, per 10 years increase)* | -0.16 (-1.22 to +0.90) | .77 |  |  |
| **Current CD4 cell count** ^D^  *(absolute, per 100 cells/mm^3^ increase)* | +0.12 (-0.23 to +0.48) | .49 |  |  |
| **Current CD8 cell count** ^D, E^  *(absolute, per 100 cells/mm^3^ increase)* | -0.12 (-0.30 to +0.07) | .22 |  |  |
| **Current CD4/8 ratio** ^D, F^  *(absolute, per 0.1 increase)* | +0.11 (-0.10 to +0.32) | .29 |  |  |

Values represent regression coefficients of linear regression with 95% confidence interval. Unit is kilogram/year weight change.

Abbreviations: ABC, abacavir; ART, antiretroviral therapy; BIC, bictegravir; BMI, body mass index; Coeff, regression coefficient; DTG, dolutegravir; EFV, efavirenz; EVG, elvitegravir; INSTI, integrase strand transfer inhibitor; NNRTI, nonnucleoside reverse transcriptase inhibitor; P, p-value; PI, protease inhibitor; RAL, raltegravir; TAF, tenofovir alafenamide; TDF, tenofovir disproxil fumarate.

A. The only variable significantly associated in backwards-stepwise selection was years since HIV diagnosis. B. At moment of discontinuation TAF/INSTI. C. BMI was categorized as underweight (<18.5 kg/m^2^), normal weight (18.5 to 24.9 kg/m^2^), overweight (25.0 to 29.9 kg/m^2^) and obese (≥30.0 kg/m^2^). D. Last known value prior to moment of discontinuation TAF/INSTI. E. Current CD8 cell count missing in 10/115. F. Current CD4/8 ratio missing in 7/115.
